# A case of Alport syndrome with pregnancy-related atypical hemolytic uremic syndrome and crescentic glomerulonephritis 

**DOI:** 10.5414/CNCS110617

**Published:** 2021-03-17

**Authors:** Ilay Berke Mentese, Murat Tugcu, Ismail Nazli, Deniz Filinte, Ebru Asicioglu, Hakki Arikan, Serhan Tuglular, Arzu Velioglu

**Affiliations:** 1 Department of Nephrology,; 2 Department of Internal Medicine, and; 3 Department of Pathology, Marmara University School of Medicine, Istanbul, Turkey

**Keywords:** pregnancy, Alport syndrome, acute kidney injury, crescentic glomerulonephritis, atypical hemolytic uremic syndrome

## Abstract

Kidney function may be impaired during pregnancy due to various reasons, and the physiological changes of pregnancy may unmask or worsen pre-existing kidney disease. Herein, we report a pregnant patient presenting with nephrotic-range proteinuria. She later developed acute kidney injury and pre-eclampsia. However, hemolytic anemia and thrombocytopenia persisted after delivery, and she was diagnosed with atypical hemolytic uremic syndrome (aHUS). Although hematological abnormalities resolved with eculizumab treatment, her renal functions did not improve. Kidney biopsy showed crescentic glomerulonephritis without thrombotic microangiopathy features. Concurrently, she was evaluated for hearing impairment, and a diagnosis of Alport syndrome was confirmed with genetic testing. Kidney function may worsen in patients with Alport syndrome during pregnancy. However, crescentic glomerulonephritis (GN) is a rare finding in Alport disease. Pauci-immune crescentic GN has been shown to be related to dysregulated activation of the alternative complement pathway, which is also the underlying pathophysiological mechanism in aHUS.

## Introduction 

Chronic kidney disease (CKD) in pregnant patients can be complicated due to the increased physiological demands on the kidney and the risk of disease progression. In the peri- and postpartum period, thrombotic microangiopathy (TMA) is one of the most significant disorders that can lead to rapid clinical deterioration. It can manifest as HELLP (hemolysis, elevated liver enzymes, low platelet count syndrome), pre-eclampsia with severe features, thrombotic thrombocytopenic purpura (TTP), or atypical hemolytic uremic syndrome (aHUS). 

On the other hand, little is known about the pregnancy course in women with Alport syndrome (AS) since it is a rare genetic disease, and the vast majority of women with AS have subtle clinical features. There is only one other case reporting AS complicated by aHUS and pauci-immune crescentic glomerulonephritis (GN) [1]. 

Here we present a unique case of a pregnant woman with aHUS showing crescentic GN features on biopsy concurrent with a new diagnosis of AS. 

## Case 

A 28-week pregnant, 22-year-old woman was admitted to our clinic because of generalized edema that had worsened over the last 10 days. The patient had been well until ~ 12 weeks before the current admission, when swelling developed in both legs. She was not on any medication except occasional oral iron therapy. There was no history of alcohol, tobacco, or illicit drug use. Her medical history was notable only for bilateral hearing impairment of unknown cause requiring hearing device since childhood. There was no family history of renal, thromboembolic, or autoimmune disease. 

On examination, temperature was 36.2 °C, heart rate 80 beats per minute, blood pressure 150/90 mmHg, respiratory rate 18 per minute, and oxygen saturation was 98% while on ambient air. The patient was alert and oriented; there was periorbital edema, diffuse crackles at the lung bases, 3+ pitting edema in both legs, and the abdomen was distended with no tenderness. Initial laboratory results revealed proteinuria on urinalysis, and isomorphic erythrocytes, granular casts, and leukocyte casts were seen in microscopic urine examination. The 24-hour urine protein excretion was 11 g. Other laboratory results are shown in Table 1. She was started on methyldopa treatment for new-onset hypertension. During follow-up, serum creatinine levels and blood pressure increased despite maximum doses of hypertensive agents and eventually, an emergency c-section was performed due to severe pre-eclampsia at 30 weeks’ gestation. 

Hypoalbuminemia, edema, and serum creatinine levels did not improve after delivery; serum lactate dehydrogenase (LDH) levels were elevated, anemia and thrombocytopenia developed. Liver function tests were normal, and she did not have any neurological signs or symptoms. Further work-up for TMA revealed a negative Coombs test, normal prothrombin time, activated thromboplastin time, international normalized ratio, and fibrinogen levels, occasional schistocytes on peripheral smear and, a normal ADAMTS13 level. Serological studies were unremarkable, with normal C3 and C4 levels and negative anti-nuclear antibody (ANA), anti-myeloperoxidase (anti-MPO) antibody, anti-proteinase3 (anti-PR3) antibody, anti-glomerular basement membrane (anti-GBM) antibodies, hepatitis B, hepatitis C, and HIV. Based on these findings, she was diagnosed with aHus. Genetic screening for aHUS revealed homozygotic polymorphism on complement factor H (CFH): c.1204C>T and heterozygotic polymorphisms on CFH c.2808G>T and c.3148A>T. 

She received 4 sessions of plasmapheresis therapy, and eculizumab treatment was started subsequently. She received hemodialysis due to symptomatic hypervolemia, which was unresponsive to diuretics. Despite improvement in thrombocytopenia and anemia, serum creatinine levels continued to rise, and her urine output decreased. A kidney biopsy was performed. 

**Figure 1:**
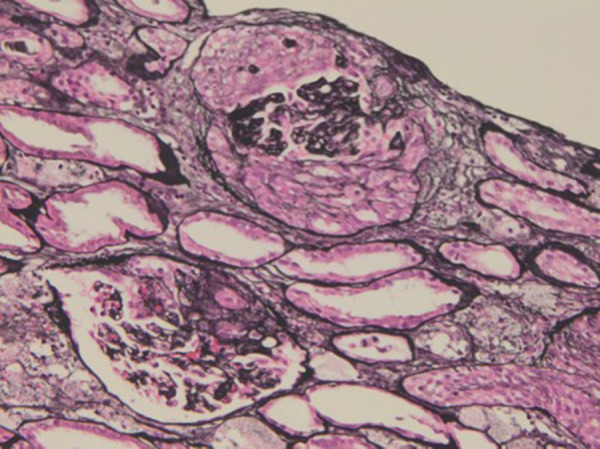
Light microscopy. Partial and circumferential crescents with focal tubular atrophy (periodic acid methenamine silver, original magnification × 40).

The sample for light microscopy showed 15 glomeruli, 11 of which had circumferential cellular crescents, and 4 had partial cellular crescents. Focal tubular atrophy was seen with sparse interstitial inflammation. No vasculitis or overt thrombosis were identified. The interlobular arteries and arterioles were well preserved. Immunofluorescence revealed 1 glomerulus, and no staining was seen with IgG, IgA, IgM, C3, C1q, κ, and λ. Electron microscopy could not be performed due to technical reasons. Based on these findings, the renal biopsy was consistent with crescentic glomerulonephritis (Figure 1). 

Further evaluation of hearing impairment revealed bilateral sensorineural hearing loss. Ophthalmic examination also revealed anterior lenticonus. Genetic screening for AS revealed a homozygous mutation on the COL4A5 gene. 

The clinical, laboratory, and genetic findings indicate a diagnosis of aHUS coexistent with AS and crescentic glomerulonephritis. Eculizumab therapy was continued, however, her kidney function did not improve. Six months after discharge, she was still on hemodialysis, at which time eculizumab treatment was stopped because of inefficacy. During follow-up, there were no complications related to the therapy. Currently, the patient is still on hemodialysis and is a kidney transplant candidate. 

## Discussion 

Alport syndrome is a genetic disease that occurs with the triad of hematuria, sensorineural hearing loss, and ocular symptoms due to the defect in the synthesis of α3, α4, and α5 chains of type 4 collagen, which is found in the basement membrane. Mutations in the *COL4A5*, *COL4A3*, or *COL4A4* genes are detected. More than 500 mutations have been shown, and the disease can be inherited in an X-linked (*COL4A5* mutations, 85% of patients), or autosomal recessive (*COL4A3* and *COL4A4* mutations, 15% of patients) fashion. In a very small group of patients, an autosomal dominant form with a better prognosis has been reported [2]. In our case, *COLA4* homozygous mutation was detected. 

Data on pregnancy outcomes in AS is limited. However, increase in proteinuria, hypertension, and the presence of pre-existing CKD have been shown to have an adverse effect on both maternal and fetal health in pregnant women with AS [3]. Brinuni et al. [4] reported a series of 6 patients where 1 patient presented only with microscopic hematuria and had no complications during the pregnancy course, but complications such as hypertension, increased proteinuria, edema, premature rupture of membranes, pre-eclampsia, or worsening of renal functions occurred in patients with proteinuria. Furthermore, 2 patients with twin pregnancies developed worsening of hypertension, nephrotic proteinuria, and pre-eclampsia, one of which had underlying advanced CKD and required renal replacement therapy [4]. Moreover, Matsuo et al. [5] reported a pregnant patient with Alport syndrome with advanced CKD, whose renal failure progressed to the end-stage with pre-eclampsia. Similarly, Yefet et al. [6] reported a patient with pre-eclampsia who eventually started peritoneal dialysis treatment. The increase in proteinuria in pregnant women with AS may be related to the fact that the physiological hyperfiltration of pregnancy has a greater effect on the defective basement membrane. Our patient presented with proteinuria and developed nephrotic syndrome, pre-eclampsia, and renal failure during follow-up, which is in accordance with the current literature. 

Typical light microscopy appearance of AS is unremarkable in early stages, however, it may show secondary glomerulosclerosis, interstitial fibrosis, and prominent interstitial foam cells later in the disease course. Electron microscopy typically shows irregular thinning and thickening glomerular basement membranes with lamellated and basket-weave appearance. However, in our case, the kidney biopsy showed crescentic glomerulonephritis, which is not a classical finding of AS. Ryu et al. [7] examined renal biopsies of 665 AS patients and demonstrated that glomerular crescents were observed in 0.4% of them, and only 3 – 11% of the glomeruli had crescent formation. In our patient, more than 90% of glomeruli had crescent formation. Haldar and Jeloke [8] also reported a young male patient with AS and crescentic GN features on biopsy. Crescent formation in AS has been hypothesized to develop as a result of the inability of the glomerular basement membrane – which has defective type 4 collagen – to maintain its structural integrity with the increase of intraglomerular pressure and capillary loop rupture [9]. As per data to date, crescents in Alport syndrome are rare but could be an additional biopsy feature suggesting faster progression and poor prognosis. 

There is increasing knowledge that the complement system is also involved in the pathogenesis of pauci-immune crescentic GN [10]. In a study conducted in anti-MPO IgG transferred rats, it was found that complement system activation is required for the development of crescents in these rats, whereas those with alternative complement component factor B and c3 deficiencies did not develop crescentic GN [11]. High plasma and urine c3a, c5a, c5b-9, Bb, and low plasma properdin levels were found to show alternative complement activation and correlate with disease activity in pauci-immune crescentic GN patients [12, 13]. Furthermore, renal biopsies positive for C3d and/or properdin showed more cellular crescents and less normal glomeruli compared to biopsies negative for C3d and/or properdin in ANCA-associated vasculitis patients [14]. 

Typically, aHUS also occurs as a result of dysregulated activation of the alternative complement pathway. The association of aHUS and pauci-immune crescentic GN has rarely been reported. Recently, Mehmood et al. [15] reported a female patient presenting with ANCA-negative pauci-immune crescentic GN who developed aHUS a month later [15]. They hypothesized that due to vasculitis, ANCA antibodies from circulation deposit in the glomerular vessels caused damage to vascular endothelium while exposing the hidden antigens and exacerbating another immune process in the form of aHUS. In a paper by Manenti et al. [16], 27% of patients with ANCA-associated vasculitis had histologic signs of TMA on the kidney biopsy, which was more frequent in patients with low C3 levels, and these patients had worse kidney survival. Therefore, anti-complement therapies might be an option in pauci-immune crescentic GN cases [17]. We hypothesize that the polymorphisms in the complement genes may have induced crescent formation readily in the presence of pregnancy and abnormal glomerular structure due to AS in our case. 

## Conclusion 

Here we presented a case with AS complicated by aHUS and crescentic glomerulonephritis. Given the complex combination of rare causes of acute kidney injury, detailed clinical evaluation is of great importance in the evaluation of acute kidney injury during pregnancy. 

## Funding 

No funding was received to assist with the preparation of this manuscript. 

## Conflict of interest 

None declared. 

**Table 1.** Laboratory data.

Variable	Reference values	On admission	At delivery	10 days post-partum	20 days post-partum	On discharge	6 months post-admission
Hemoglobin (g/dL)	12 – 17	9.2	7.8	6.9	7.2	9.5	9.3
White cell count (10^3^/μL)	4 – 10	8.3	5.7	8	6.3	6.2	4.4
Platelet count (10^3^/μL)	150 – 440	147	136	84	109	125	199
Reticulocyte count (%)	0.5 – 2.5%	1.5	–	–	2.7	2.9	–
Peripheral blood smear	100/HPF	1 – 2 schistocytes, 10 – 11 platelets, 9 – 10 echinocytes	1 – 2 schistocytes, 11 – 12 platelets	5 – 6 schistocytes, 9 – 10 platelets, 10 – 12 echinocytes	1 – 2 schistocytes, enough platelets	3 – 4 schistocytes, 12 – 13 platelets	–
Haptoglobin (g/L)	0.32 – 1.97	1.16	–	–	–	1.32	1.4
Blood urea nitrogen (mg/dL)	6 – 23	17	22	30	33	92	59
Creatinine (mg/dL)	0 – 1.2	0.78	1.1	3.2	4.81	3.04	7.19
Albumin (mg/dL)	3.5 – 5.4	1.6	1.5	2.4	2.5	2.7	4.3
Alanine aminotransferase (U/L)	10 – 40 U/L	12	9	14	18	13	27
Aspartate aminotransferase (U/L)	10 – 37	18	18	16	22	28	29
Alkaline phosphatase (U/L)	30 – 120	31	37	63	–	66	50
Total bilirubin (mg/dL)	0 – 1.3	0.15	0.2	–	0.5	0.32	0.46
Direct bilirubin (mg/dL)	0 – 0.3	0.02	0.04	–	–	0.06	0.09
Lactate dehydrogenase (U/L)	0 – 248 U/L	141	319	376	340	325	232
Uric acid (mg/dL)	3.4 – 7	5.89	6.6	7.4	6.53	4.54	5.3
24-hour urine protein excretion (mg/day)	0 – 150	11,059	–	–	18,270	8,401	–

## References

[1] Tao J Lieberman J Lafayette RA Kambham N A rare case of Alport syndrome, atypical hemolytic uremic syndrome and Pauci-immune crescentic glomerulonephritis. BMC Nephrol. 2018; 19: 355. 30541482 10.1186/s12882-018-1170-4 PMC6291978

[2] Heidet L Gubler MC The renal lesions of Alport syndrome. J Am Soc Nephrol. 2009; 20: 1210–1215. 19470679 10.1681/ASN.2008090984

[3] Alessi M Fabris A Zambon A Cremasco D Muraro E Dosa L Anglani F Del Prete D Pregnancy in Alport syndrome: a report of two differently-evolving cases. J Obstet Gynaecol. 2014; 34: 98–100. 24359068 10.3109/01443615.2013.834299

[4] Brunini F Zaina B Gianfreda D Ossola W Giani M Fedele L Messa P Moroni G Alport syndrome and pregnancy: a case series and literature review. Arch Gynecol Obstet. 2018; 297: 1421–1431. 29492669 10.1007/s00404-018-4720-x

[5] Matsuo K Tudor EL Baschat AA Alport syndrome and pregnancy. Obstet Gynecol. 2007; 109: 531–532. 17267885 10.1097/01.AOG.0000240141.26395.82

[6] Yefet E Tovbin D Nachum Z Pregnancy outcomes in patients with Alport syndrome. Arch Gynecol Obstet. 2016; 293: 739–747. 26411580 10.1007/s00404-015-3893-9

[7] Ryu M Migliorini A Miosge N Gross O Shankland S Brinkkoetter PT Hagmann H Romagnani P Liapis H Anders HJ Plasma leakage through glomerular basement membrane ruptures triggers the proliferation of parietal epithelial cells and crescent formation in non-inflammatory glomerular injury. J Pathol. 2012; 228: 482–494. 22553158 10.1002/path.4046

[8] Haldar I Jeloka T Alport’s Syndrome: A Rare Clinical Presentation with Crescents. Indian J Nephrol. 2020; 30: 129–131. 32269440 10.4103/ijn.IJN_177_19 PMC7132844

[9] Chang A Logar CM Finn LS Alpers CE Seliger SL A rare cause of necrotizing and crescentic glomerulonephritis in a young adult male. Am J Kidney Dis. 2005; 45: 956–960. 15861364 10.1053/j.ajkd.2004.08.046

[10] Sethi S Zand L De Vriese AS Specks U Vrana JA Kanwar S Kurtin P Theis JD Angioi A Cornell L Fervenza FC Complement activation in pauci-immune necrotizing and crescentic glomerulonephritis: results of a proteomic analysis. Nephrol Dial Transplant. 2017; 32: i139–i145. 28391334 10.1093/ndt/gfw299

[11] Xiao H Schreiber A Heeringa P Falk RJ Jennette JC Alternative complement pathway in the pathogenesis of disease mediated by anti-neutrophil cytoplasmic autoantibodies. Am J Pathol. 2007; 170: 52–64. 17200182 10.2353/ajpath.2007.060573 PMC1762697

[12] Gou SJ Yuan J Chen M Yu F Zhao MH Circulating complement activation in patients with anti-neutrophil cytoplasmic antibody-associated vasculitis. Kidney Int. 2013; 83: 129–137. 22913983 10.1038/ki.2012.313

[13] Gou SJ Yuan J Wang C Zhao MH Chen M Alternative complement pathway activation products in urine and kidneys of patients with ANCA-associated GN. Clin J Am Soc Nephrol. 2013; 8: 1884–1891. 24115193 10.2215/CJN.02790313 PMC3817906

[14] Hilhorst M van Paassen P van Rie H Bijnens N Heerings-Rewinkel P van Breda Vriesman P Cohen Tervaert JW Complement in ANCA-associated glomerulonephritis. Nephrol Dial Transplant. 2017; 32: 1302–1313. 26275893 10.1093/ndt/gfv288

[15] Mehmood M Anees M Ahmad S Elahi I Mateen F Hussain M Ashraf S Coexistence of Anti Neutrophilic Cytoplasmic Antibody (ANCA) Negative Renal Limited Vasculitis and Atypical- Hemolytic Uremic Syndrome (aHUS). Iran J Kidney Dis. 2023; 15: 391–394. 10.52547/ijkd.6443 34582374

[16] Manenti L Vaglio A Gnappi E Maggiore U Allegri L Allinovi M Urban ML Delsante M Galetti M Nicastro M Pilato FP Buzio C Association of Serum C3 Concentration and Histologic Signs of Thrombotic Microangiopathy with Outcomes among Patients with ANCA-Associated Renal Vasculitis. Clin J Am Soc Nephrol. 2015; 10: 2143–2151. 26542163 10.2215/CJN.00120115 PMC4670755

[17] Jayne DRW Bruchfeld AN Harper L Schaier M Venning MC Hamilton P Burst V Grundmann F Jadoul M Szombati I Tesař V Segelmark M Potarca A Schall TJ Bekker P Randomized Trial of C5a Receptor Inhibitor Avacopan in ANCA-Associated Vasculitis. J Am Soc Nephrol. 2017; 28: 2756–2767. 28400446 10.1681/ASN.2016111179 PMC5576933

